# Supplementary material for: UV-curable thiol-ene system for broadband infrared transparent objects
Source: Nat Commun. 2023 Dec 16;14:8385. doi: 10.1038/s41467-023-44273-0 (PMC10725491; doi:10.1038/s41467-023-44273-0)
Supplement: Supplementary file 1 — Supplementary_Information [file 41467_2023_44273_MOESM1_ESM.docx]

**Supplementary Information**

**UV-Curable Thiol-ene System for Broadband Infrared Transparent Objects**

**Piaoran Ye,^1^****† Zhihan Hong,^1^† Douglas A. Loy,^2,3^ Rongguang Liang^1*^**

^1^Wyant College of Optical Sciences, The University of Arizona, 1630 E. University Blvd, Tucson, Arizona 85721, USA

^2^Department of Chemistry&Biochemistry, The University of Arizona, 1306 E. University Blvd, Tucson, Arizona 85721-0041, USA

^3^Department of Materials Science&Engineering, The University of Arizona, 1235 E. James E. Rogers Way, Tucson, Arizona 85721-0012, USA

^*^Corresponding author: [rliang@optics.arizona.edu](mailto:rliang@optics.arizona.edu)

† These two authors contributed equally to this work.

**Supplementary Discussion**

**Discussion on 3D printing of micro-channels**

To prove the 3D printability as well as to test the printing resolution, we used tetraSH-DVO2 to print a multi-channel structure, which was inspired by 3D micro integrated fluid system,^1^ as shown in Supplementary Figure 20.

Nowadays, most microfluidic devices are limited to two-dimensional microchannels because they were manufactured by photolithographic patterning on glass or silicon substrates and the subsequent bonding process.^2^ On the other side, 3D micro integrated fluid system is a concept that has been developed since 1990s, which contains 3D-structured microchannels and was originally developed for bio-synthetic and bio-sensing applications.^1^ Such a strategy allows a more compact design and can integrate many functions into one microfluidic system. The similar concept has been applied to fabricate integrated microfluidic or microchannel systems that has other functions including micro-cooling system,^3,4^ microsphere generation,^5^ fluidic coupler (mixer),^6^ etc. Regarding the fabrication method, 3D printing has its unique advantage in fabricating complex 3D structures and has been reported to fabricate 3D integrated microfluidics or mircochannels.^7-9^

In this study, we present the fabrication of a micro-module incorporating microchannels using our tetraSH-DVO2 material. The resulting cubic structure contains 12 x 14 microchannels, each with an approximate diameter of 10 µm (Figure S20). It's worth noting that a minor flaw in printing quality is observed in the upper right corner of the structure. This phenomenon can be attributed to its proximity to the boundary of the objective's field of view during the printing process. Our investigations reveal that this diameter represents the lower threshold achievable for micro-channels when utilizing the tetraSH-DVO2 resin. While this resolution falls below that of most TPP 3D printing methods, and there's a possibility of merging in channels with smaller diameters, this resolution limitation of the tetraSH-DVO2 resin is still suitable for numerous MWIR and LWIR applications. These applications predominantly involve IR with wavelengths surpassing 3 µm. In future work, we intend to integrate the 3D structured microchannels with IR signal monitoring. This undertaking will necessitate proper design of the structure and a comprehensive optimization of both the resin composition and printing parameters.

Supplementary Table 1. IR transparency comparison between some reported material and multi-thiol-DVO material in this work. Ge and PE are listed as references.

|  | | | Max MWIR transparency at reported thickness | Max LWIR transparency at reported thickness | Curing mechanism | Reported fabrication method |
| --- | --- | --- | --- | --- | --- | --- |
| PE  (this work) | | LDPE | 70% (500 μm) | 60% (500 μm) | - | Heat pressing |
|  |  | HDPE | 42% (500 μm) | 43% (500 μm) |  |  |
| Ge^10^ | | | ~48% (1 mm)* | ~44% (1 mm)* | - | - |
| S+DIB^11^ | | | 60% (200 μm) | - | Inverse vulcanization | Molding  (~135-~175°C) |
| S+Se+DIB^11^ | | | 60% (200 μm) | - |  |  |
| S+DVB^12^ | m-DVB | | 42% (1 mm) | 15% (1 mm) |  |  |
|  | p-DVB | | 52% (1.1 mm) | 15% (1.1 mm) |  |  |
|  | mixed-DVB | | 55% (1.69 mm) | 12% (1.69 mm) |  |  |
| S+norbornadiene^13^ | | | ~32% (1 mm) | ~25% (1 mm) |  |  |
| S+tetravinyltin^14^ | | | ~32% (1.55 mm) | ~2% (1.55 mm) |  |  |
| S+benzenetriSH^10^ | | | ~72% (1 mm) | ~72% (1 mm) | Thiol-S coupling | Molding  (185°C) |
| TriSH+trivinyl^15^ | | | 62% (200 μm) | 24% (200 μm) | Thiol-ene | Imprint  (UV) |
| tetraSH-DVO2  (this work) | | | 55% (500 μm)  31% (900 μm)  7% (2 mm) | 72% (150 μm)  16% (500 μm) |  | Molding (UV),  TPP 3D printing |
| polySH-DVO2  (this work) | | | 64% (500 μm)  30% (900 μm)  9% (2 mm) | 70% (150 μm)  20% (500 μm) |  |  |

*Unlike all the reported polymers which show discontinuous transparent windows on the spectrum, Ge shows a continuous transparent window through MWIR and LWIR region.


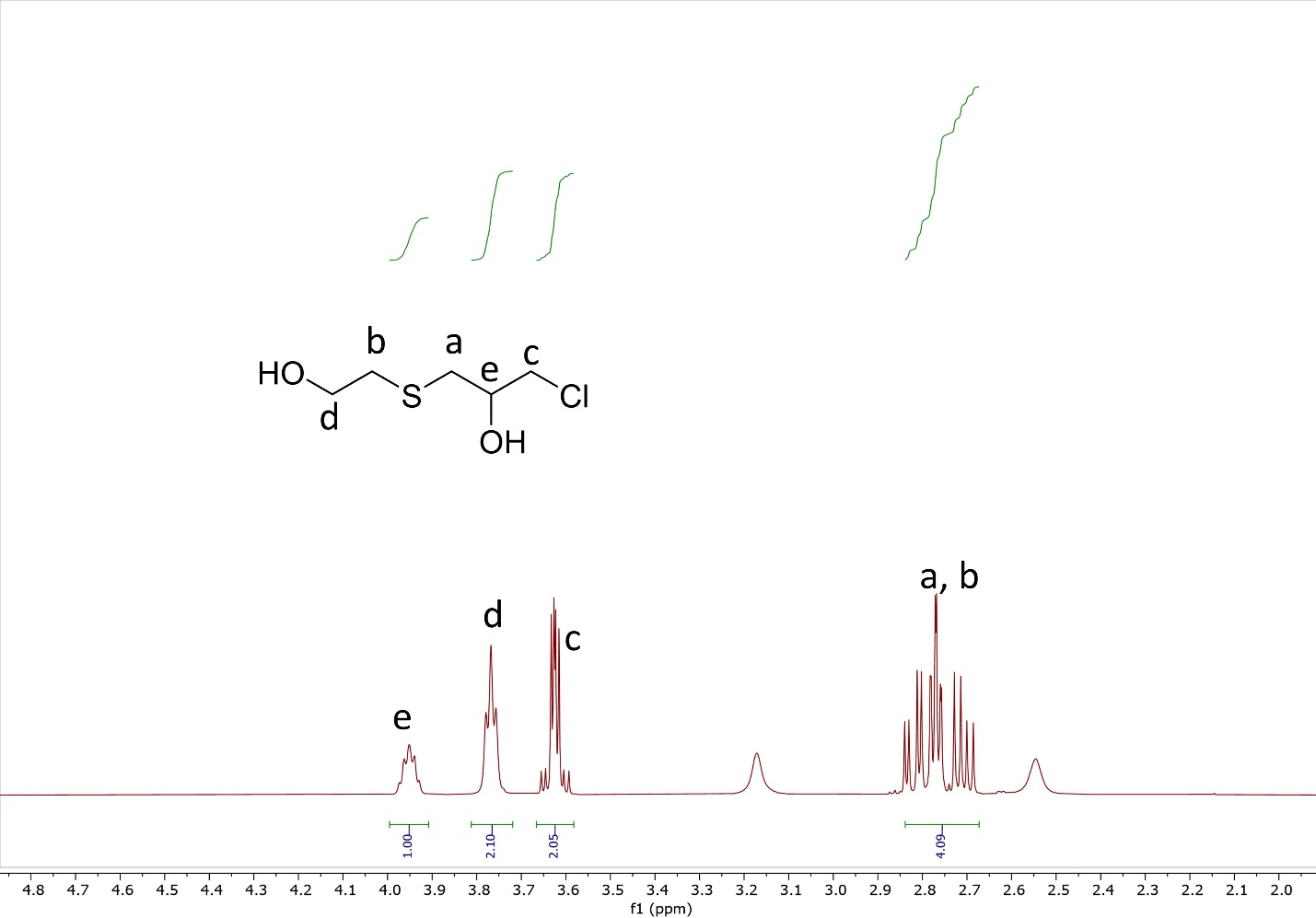


Supplementary Figure 1. ^1^H NMR of 1-chloro-3-(hydroxyethylthio)-2-propanol (CHTEP).


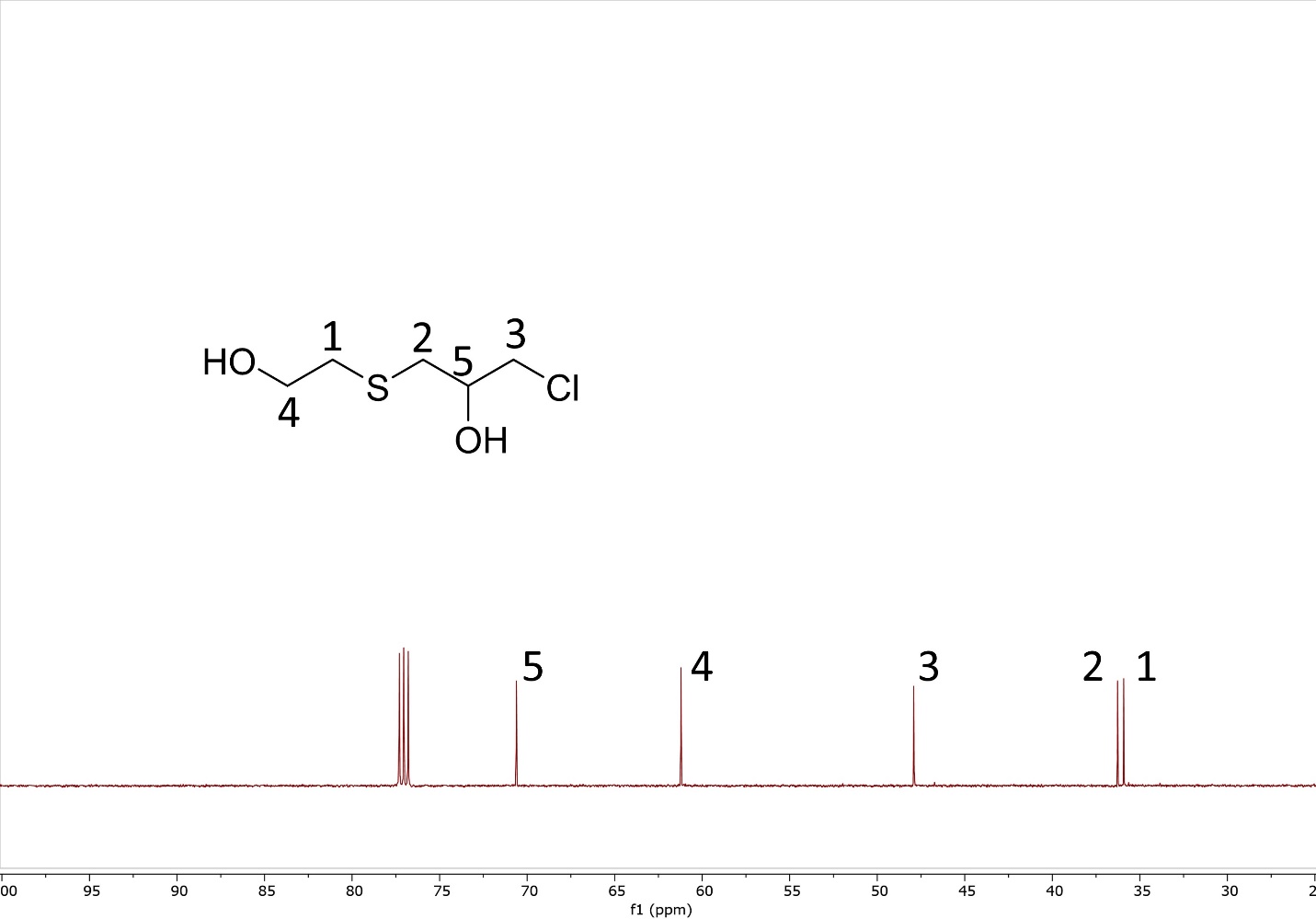


Supplementary Figure 2. ^13^C NMR of 1-chloro-3-(hydroxyethylthio)-2-propanol (CHTEP).


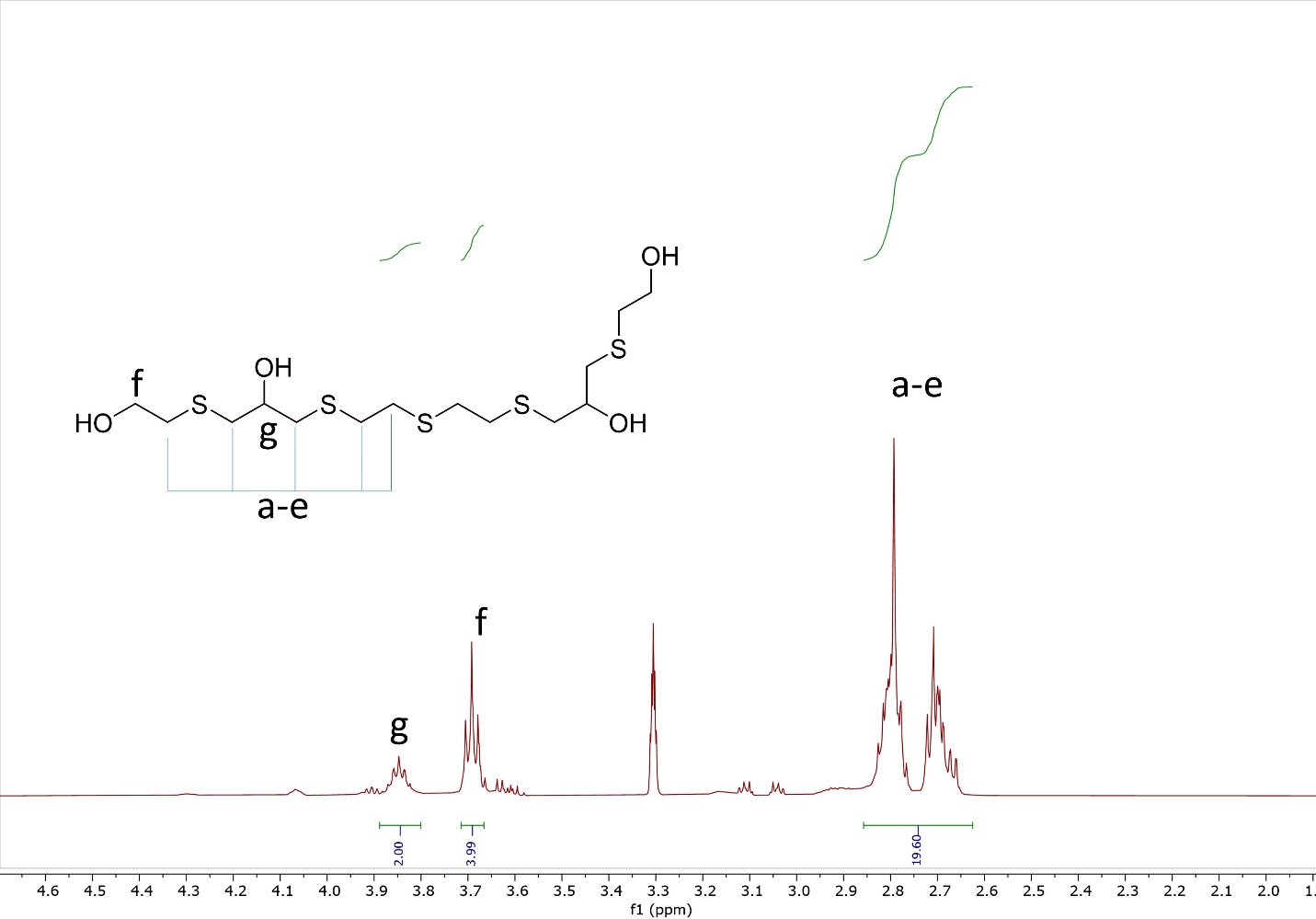


Supplementary Figure 3. ^1^H NMR of polyOH.


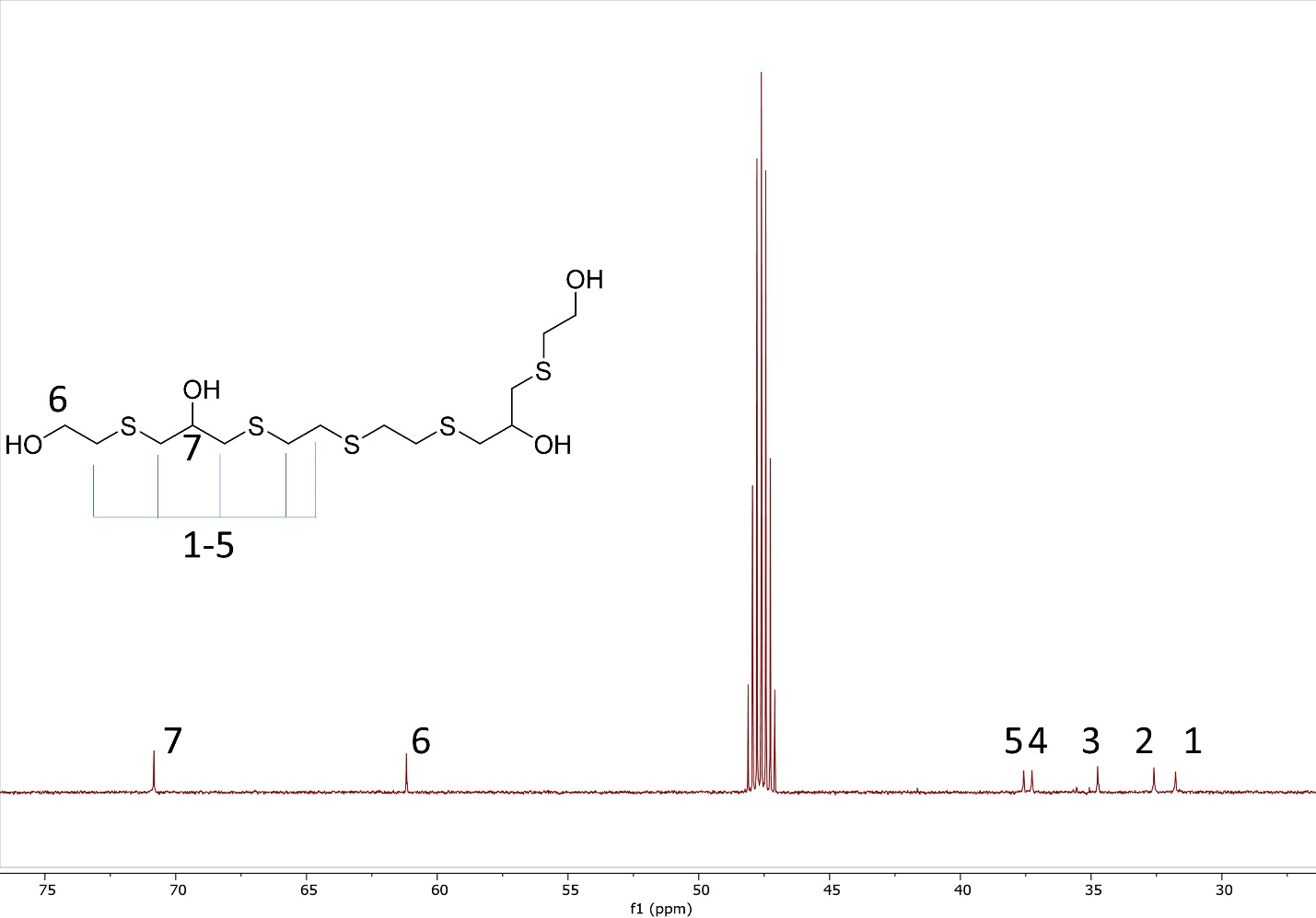


Supplementary Figure 4. ^13^C NMR of polyOH.


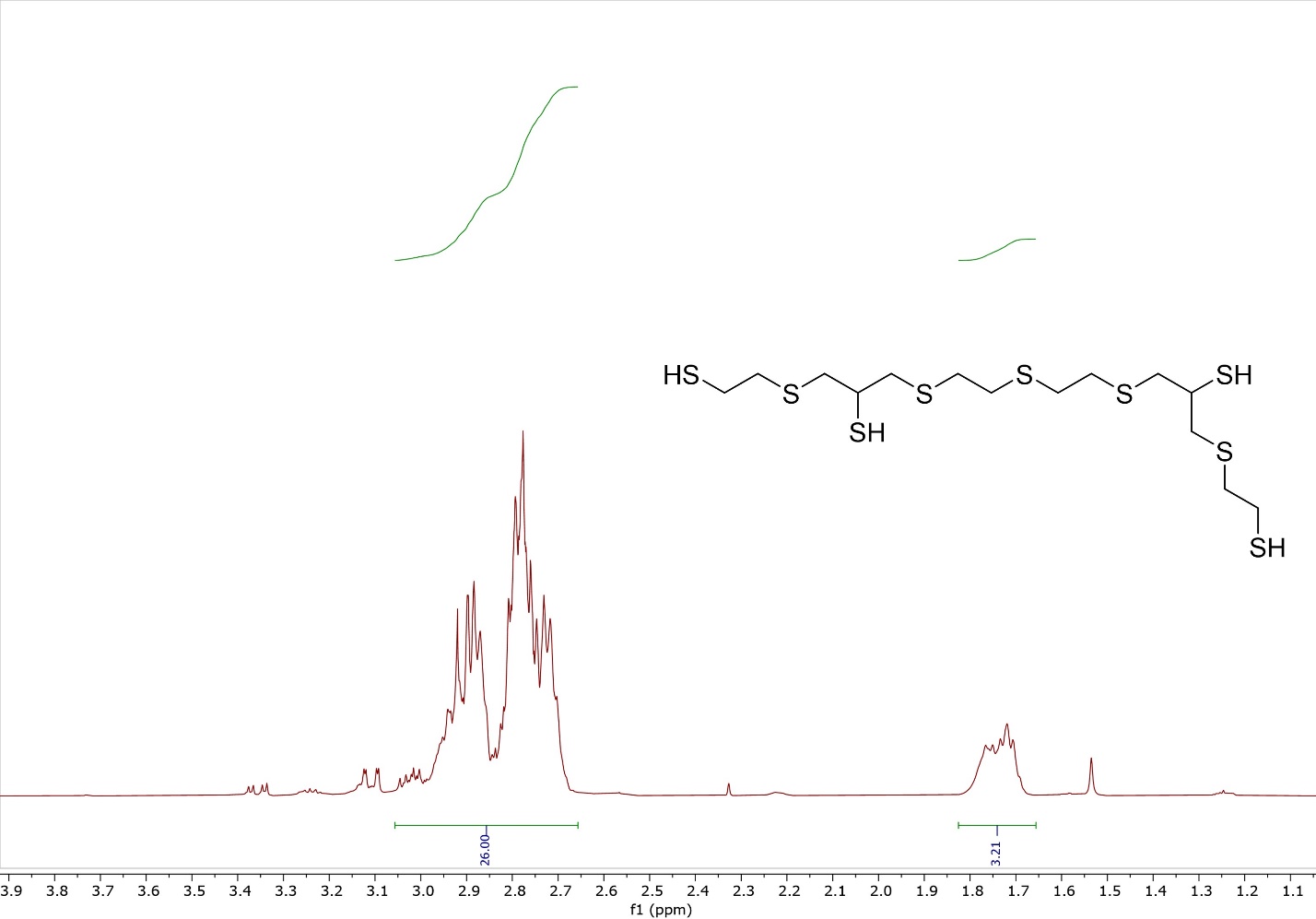


Supplementary Figure 5. ^1^H NMR of polySH. The integral of thiol peaks indicates that the final product is not pure.


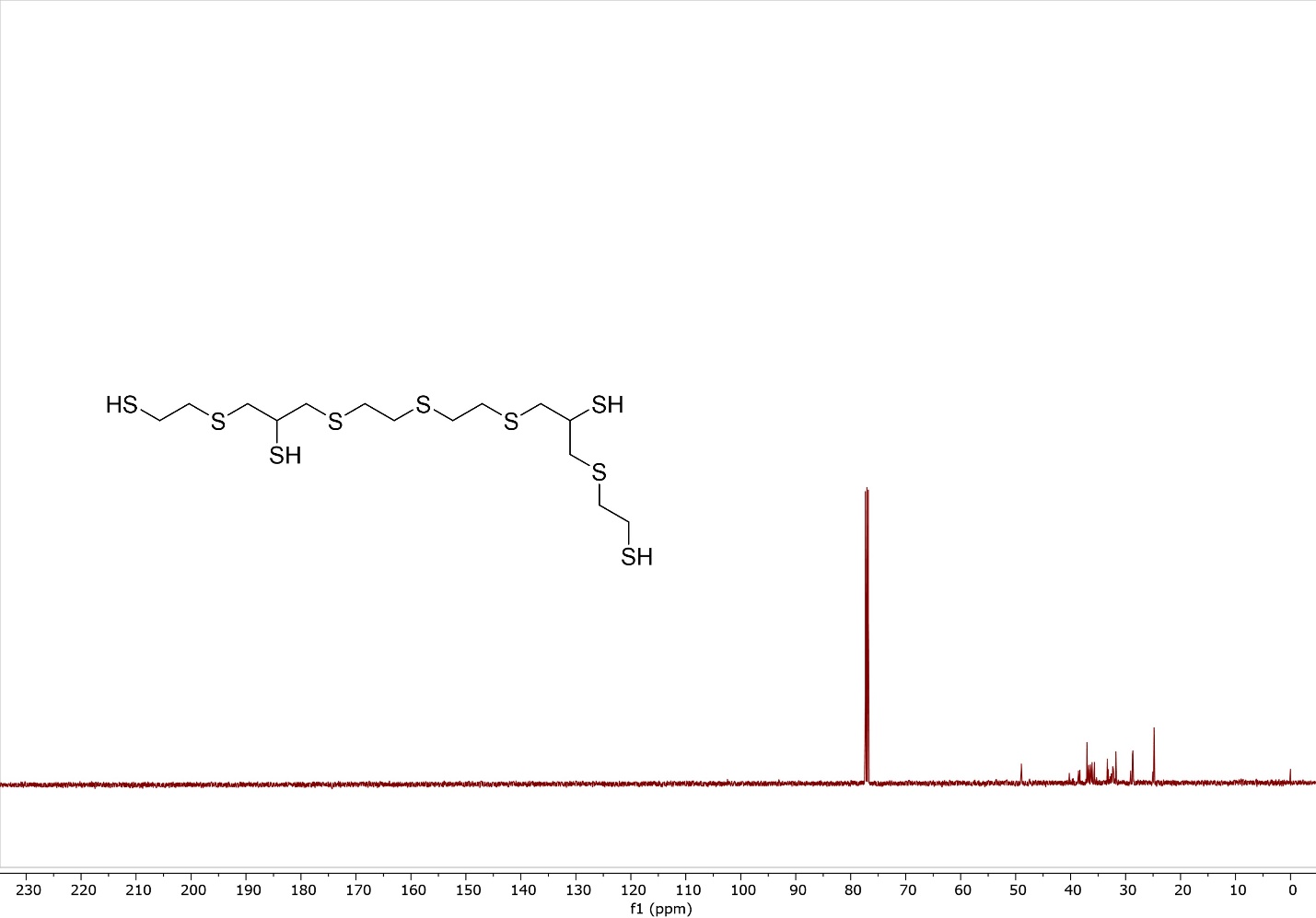


Supplementary Figure 6. ^13^C NMR of polySH


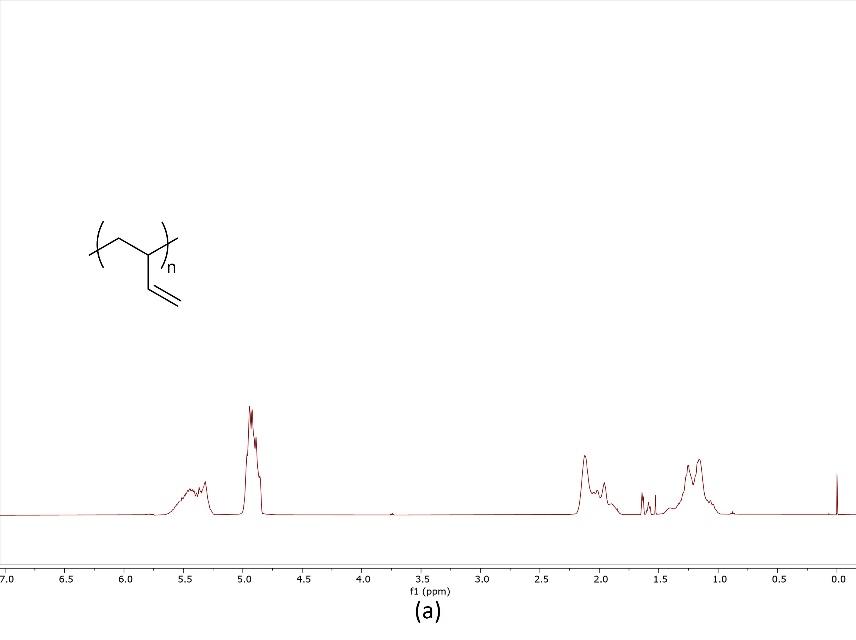

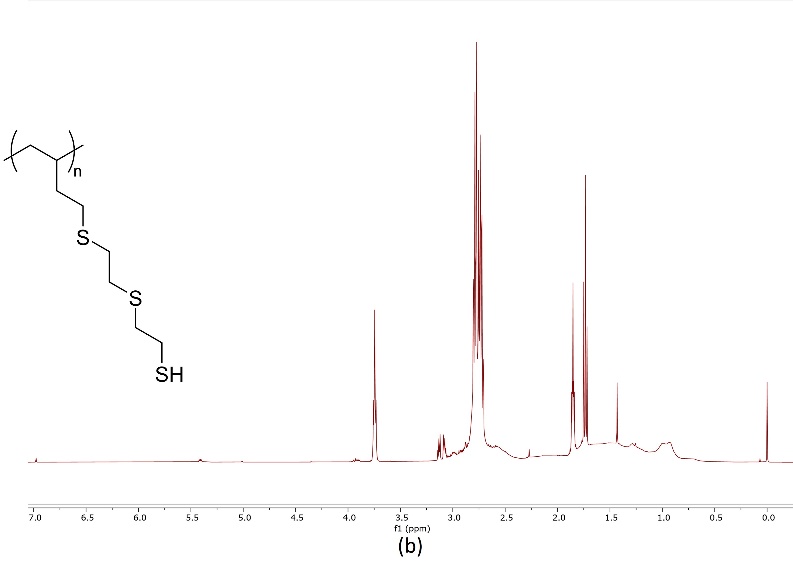

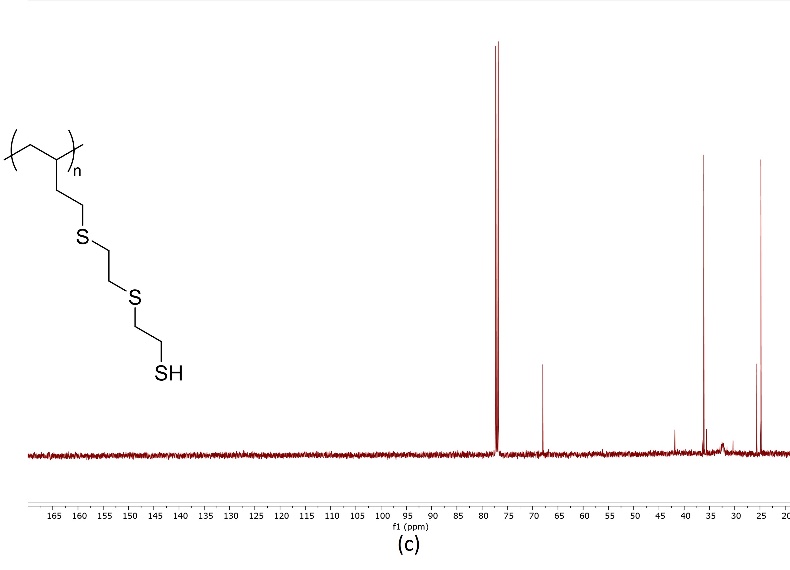


Supplementary Figure 7. (a) ^1^H NMR of polybutadiene (1,2 addition); (b) ^1^H NMR of polySH, and (c) ^13^C NMR of polySH.


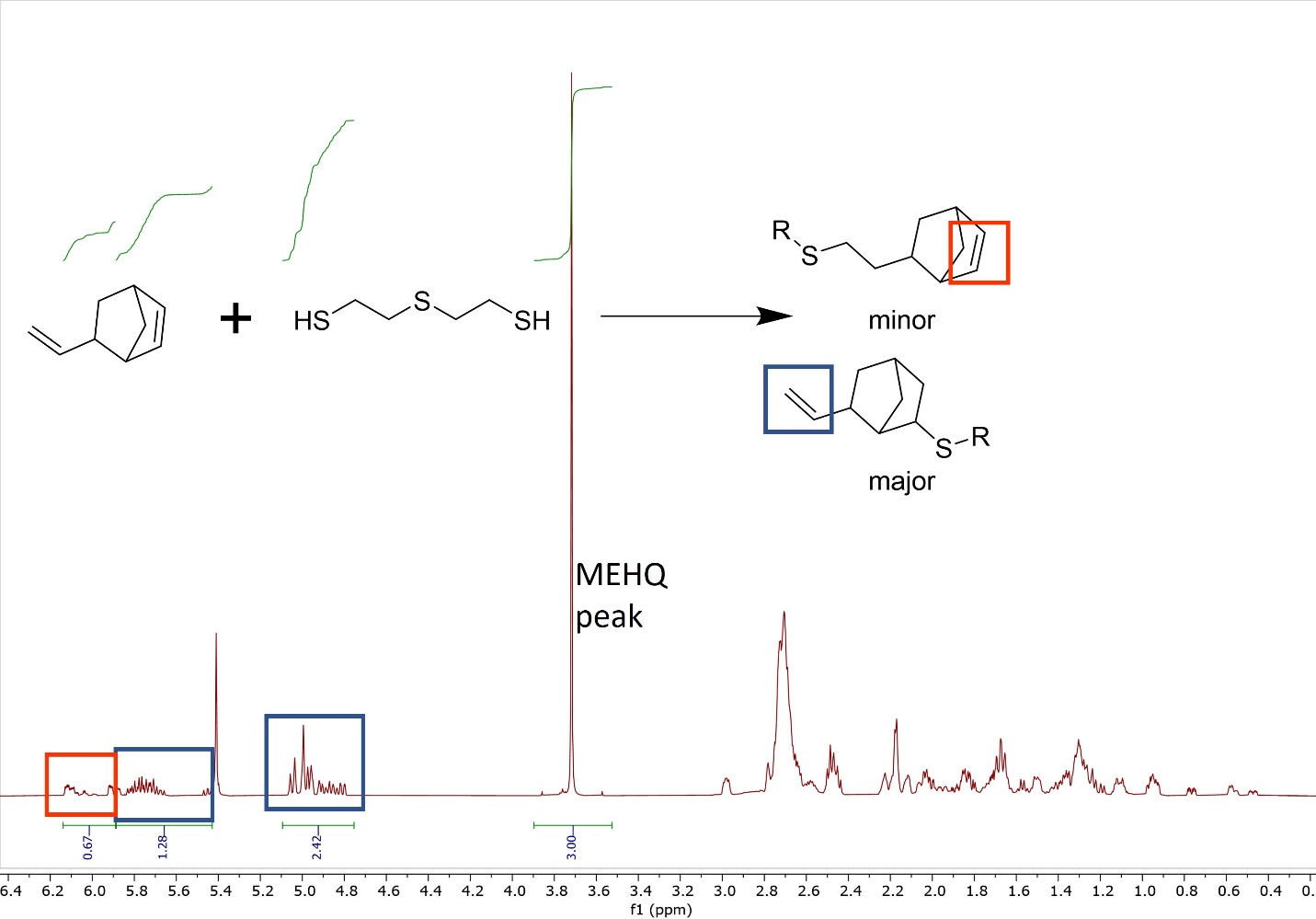


Supplementary Figure 8. ^1^H NMR of diene oligomers after the reaction between 2,2 -thiodiethanethiol and 5-vinyl-2-norbornene. The 4-methoxyphenol (MEHQ) was added to determine the C=C concentration in final products.


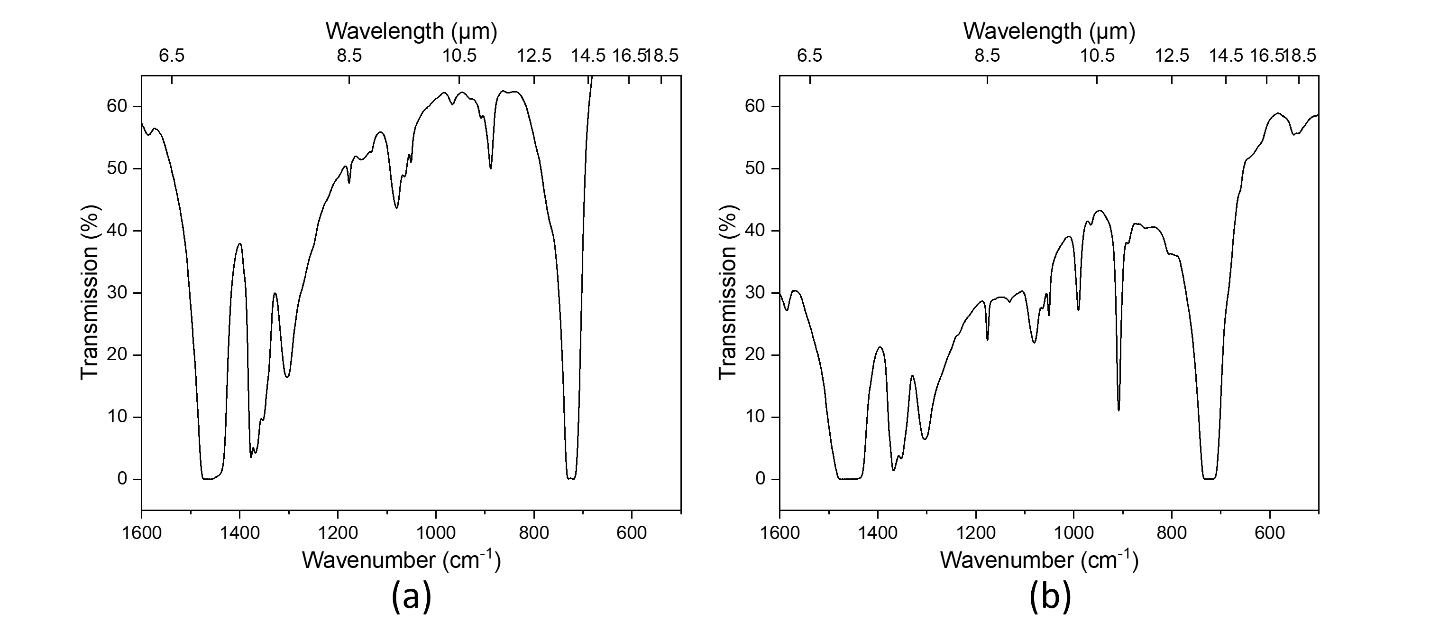


Supplementary Figure 9. (a) Long-wave infrared (LWIR) transmission of a low-density polyethylene (LDPE) sample with around 500 μm thickness, and (b) LWIR transmission of a high-density polyethylene (HDPE) sample with around 500 μm thickness. Source data are provided as a Source Data file.


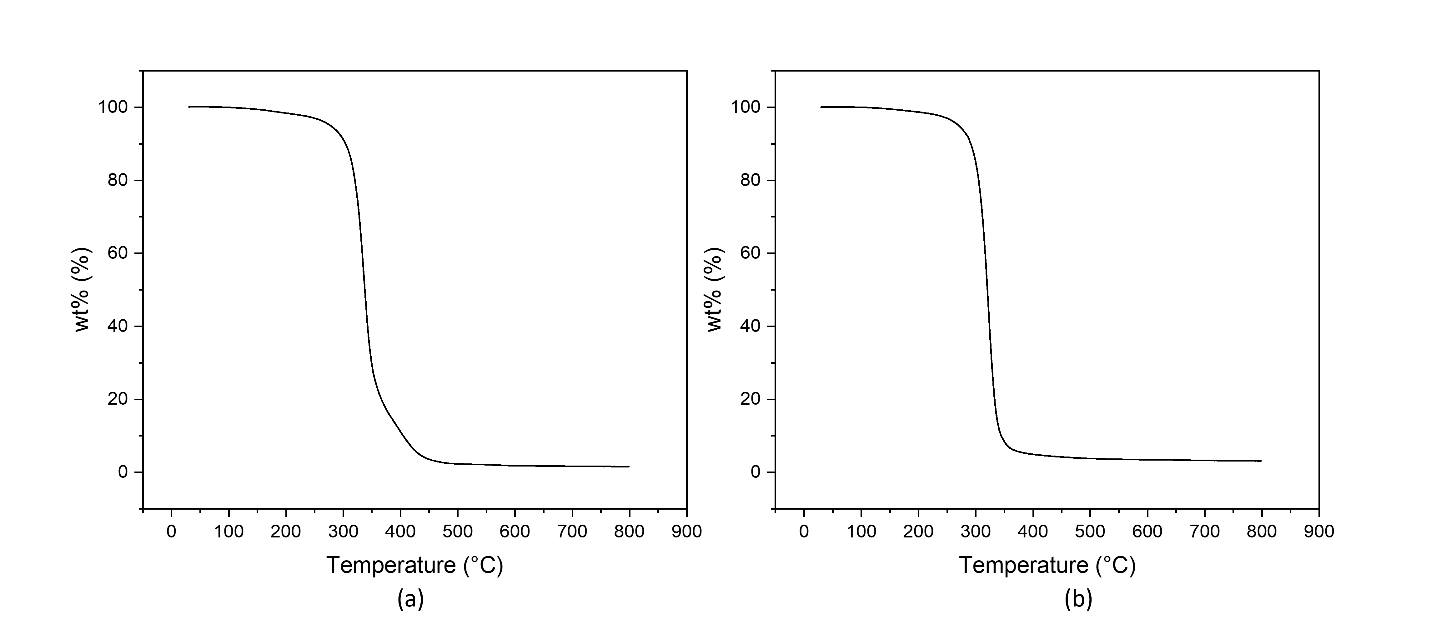


Supplementary Figure 10. Thermogravimetric Analysis (TGA) of (a) sample prepared with polySH and (b) sample prepared with tetraSH. Source data are provided as a Source Data file.


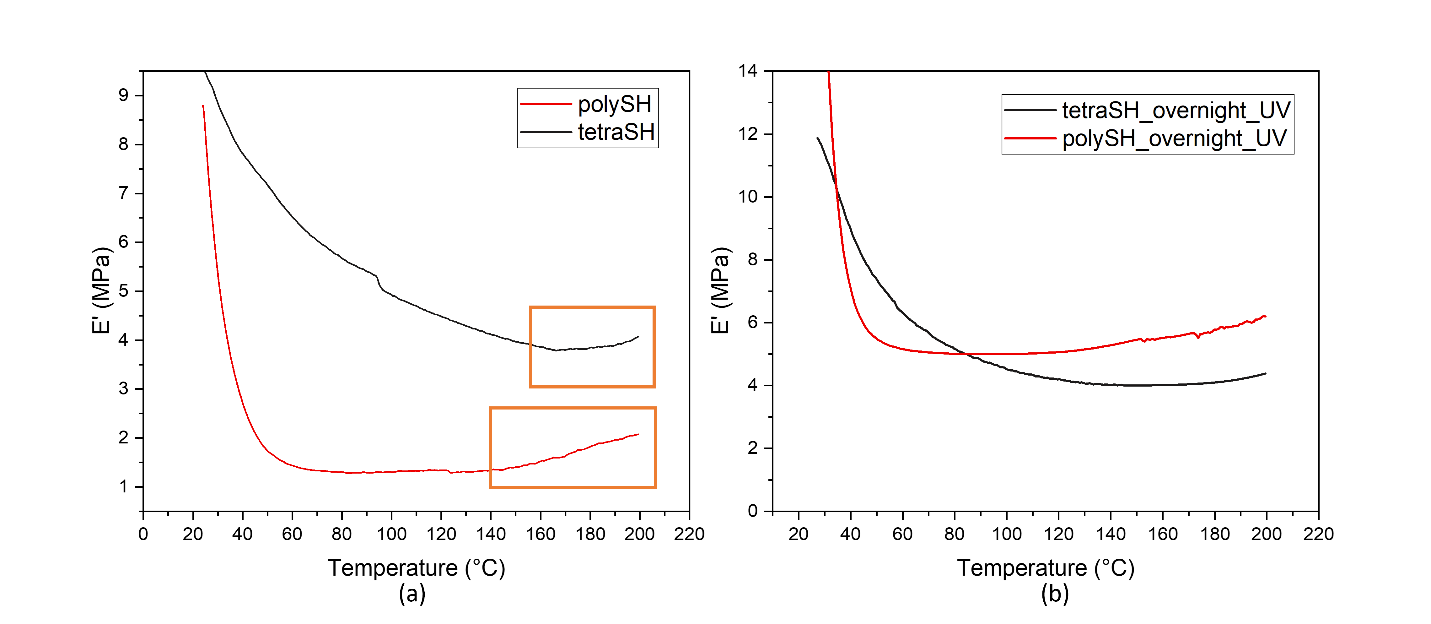


Supplementary Figure 11. Dynamic Mechanical Analysis (DMA) tests of samples prepared with polySH-DVO2 and tetraSH-DVO2. (a) The samples were cured by UV (OmniCure) for 10 minutes. (b) The samples were firstly cured by OmniCure for 10 minutes and then post cured in Formlabs curing machine for 12h. Source data are provided as a Source Data file.


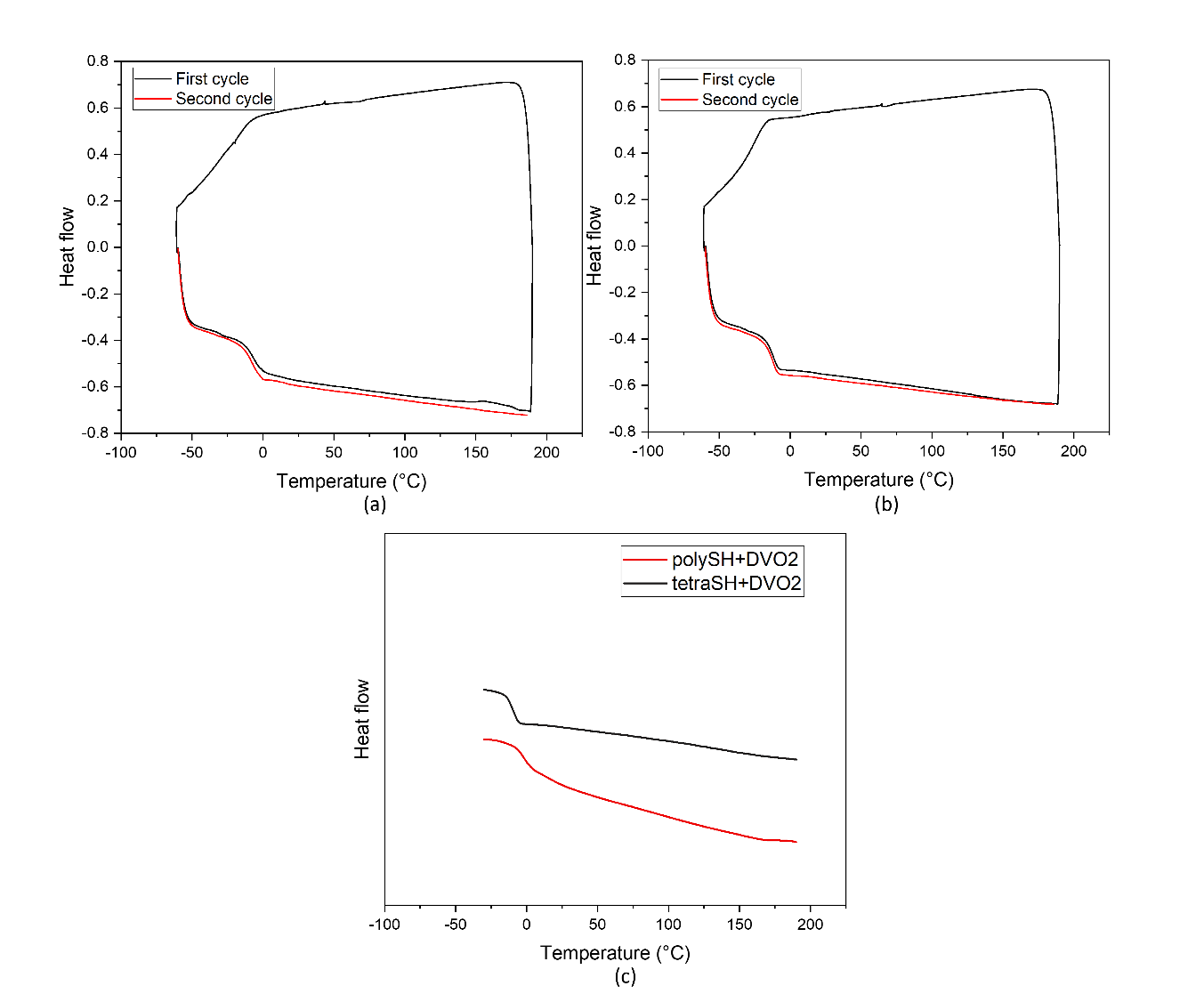


Supplementary Figure 12. Differential Scanning Calorimetry (DSC) of (a) polySH-DVO2 cured with 10 min’s UV exposure, (b) tetraSH-DVO2 cured with 10 min’s UV exposure, and (c) both sample cured with 10 min’s UV exposure and then UV post-cured in Formlabs curing machine for 12h at room temperature. Source data are provided as a Source Data file.


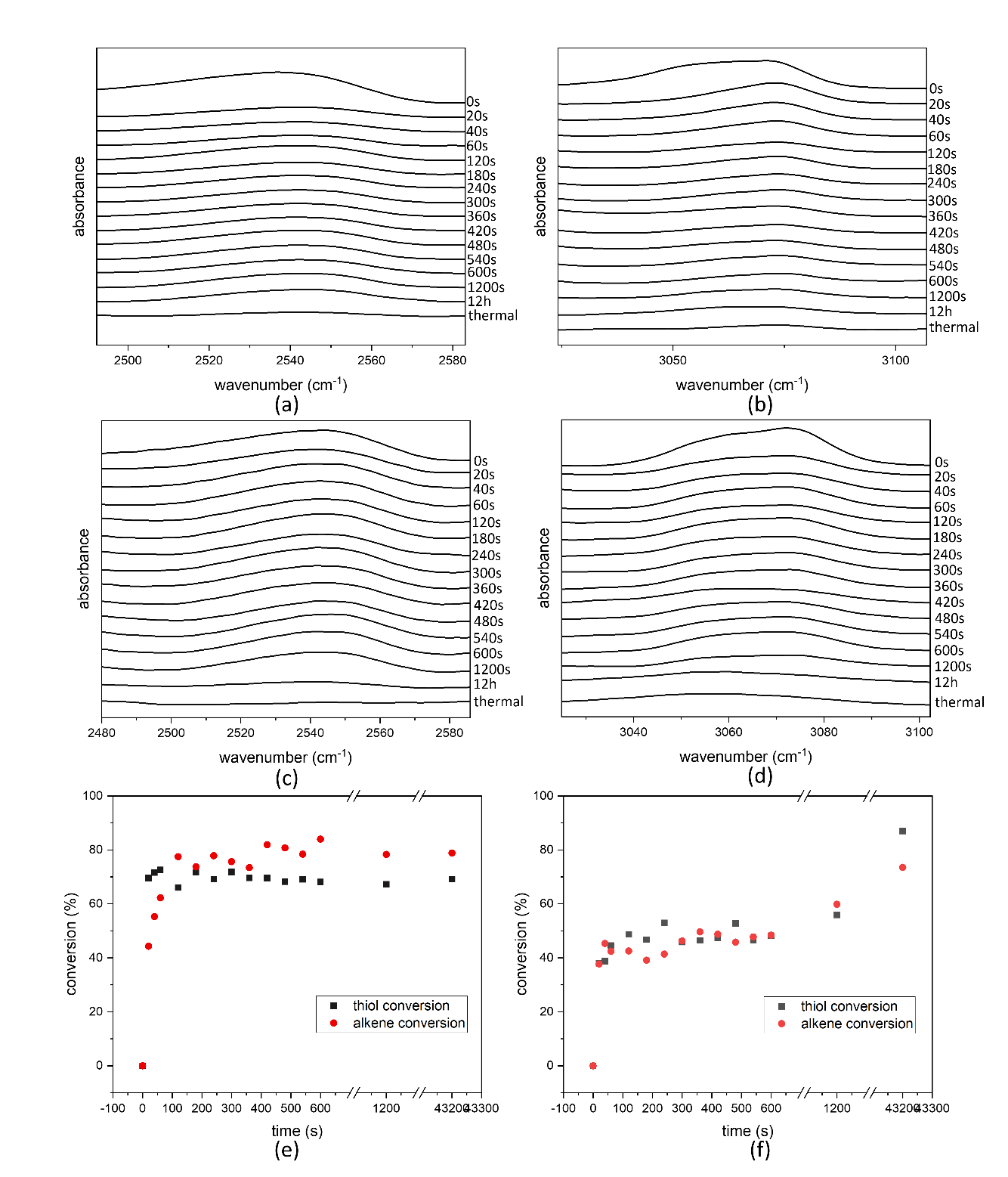


Supplementary Figure 13. Fourier Transform Infrared Spectroscopy (FTIR) and functional groups conversion of multithiol-DVO2 cured for different time. (a)-(b) FTIR-ATR spectra of tetraSH-DVO2 cured under different time. The curve named “thermal” means the samples were firstly cured under UV for 10 minutes and then thermally cured at 150 °C for 12h. (c)-(d) FTIR-ATR spectra of polySH-DVO2 cured under different time. (e) Thiol and alkene conversion of tetraSH-DVO2 system calculated from (a) and (b). (f ) Thiol and alkene conversion of tetraSH-DVO2 system calculated from (c) and (d). The first 1200s UV exposure were conducted with relatively strong irradiation and the last data point (43200s, 12h) refers to sample cured by relatively week irradiation. Source data are provided as a Source Data file.


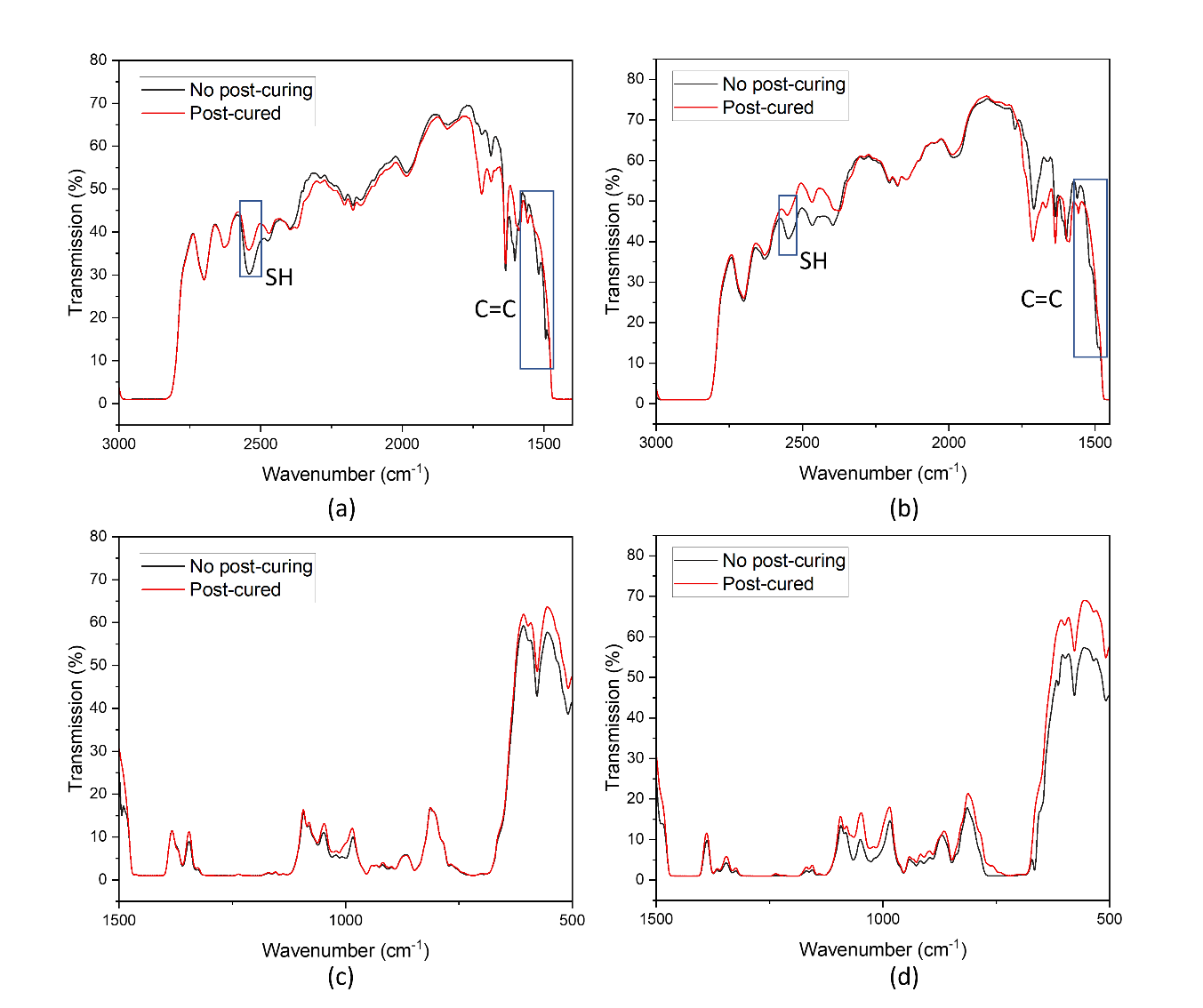


Supplementary Figure 14. FTIR comparison of samples (a) prepared with tetraSH and DVO2 (MWIR), (b) prepared with polySH and DVO2 (MWIR), (c) prepared with tetraSH and DVO2 (LWIR), and (d) prepared with polySH and DVO2 (LWIR) before and after post-curing. Source data are provided as a Source Data file.


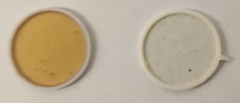


Supplementary Figure 15. Picture of samples prepared using polySH and divinyl oligomer (DVO2). The left one showing brownish color was post-cured in air and the right one was post-cured in nitrogen. The white ring outside of the sample is a 3D-printed PLA ring that helps to hold the shape during UV curing.


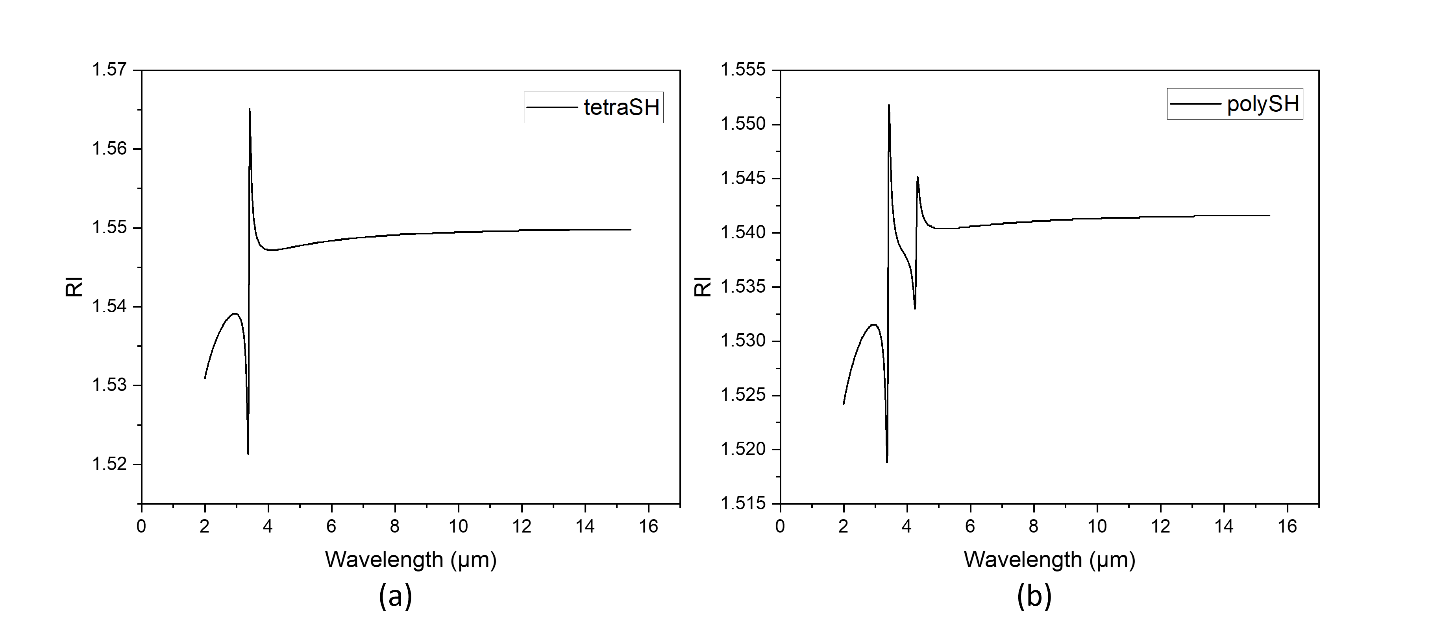


Supplementary Figure 16. The refractive index of cured multithiol-DVO2 system. (a) Thin film prepared with tetraSH and DVO2. (b) Thin film prepared with polySH and DVO2. Source data are provided as a Source Data file.


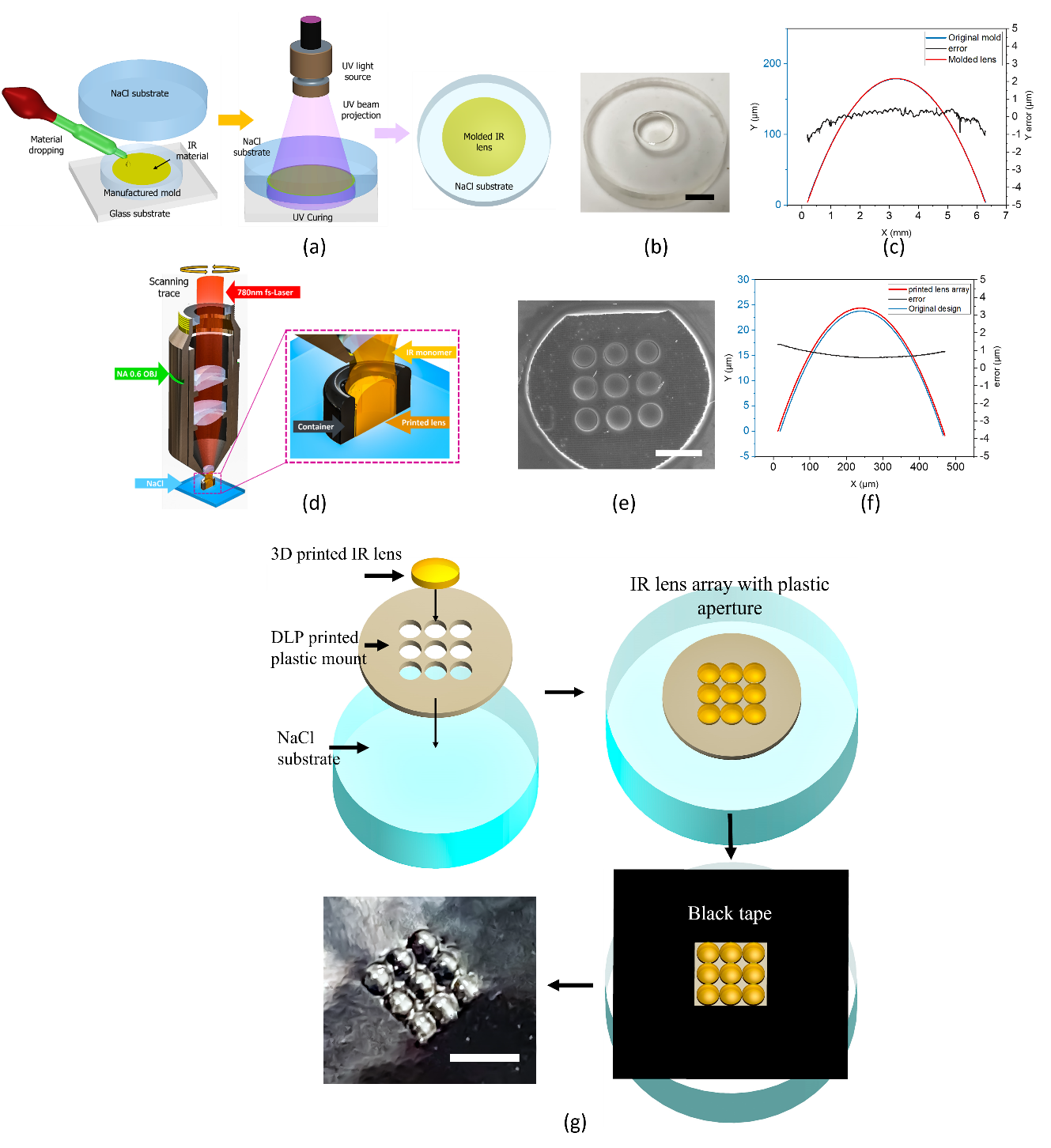


Supplementary Figure 17. Schematic workflow of the molding fabrication method and the two-photon polymerization 3D printing method. (a) The workflow of molding method. (b) The molded lens shows good transparency to visible light. Scale bar: 5 mm. (c) The comparison of the mold and the molded lens; (d) The schematic diagram of two-photon printing of IR transparent resin; (e)The 3D printed 3X3 IR lens array, scale bar = 1 mm; (f) The comparison of one singlet lens in lens array and the original design. (g) The scheme of assembling printed lens array with black mask for imaging performance evaluation. scale bar = 1 mm.


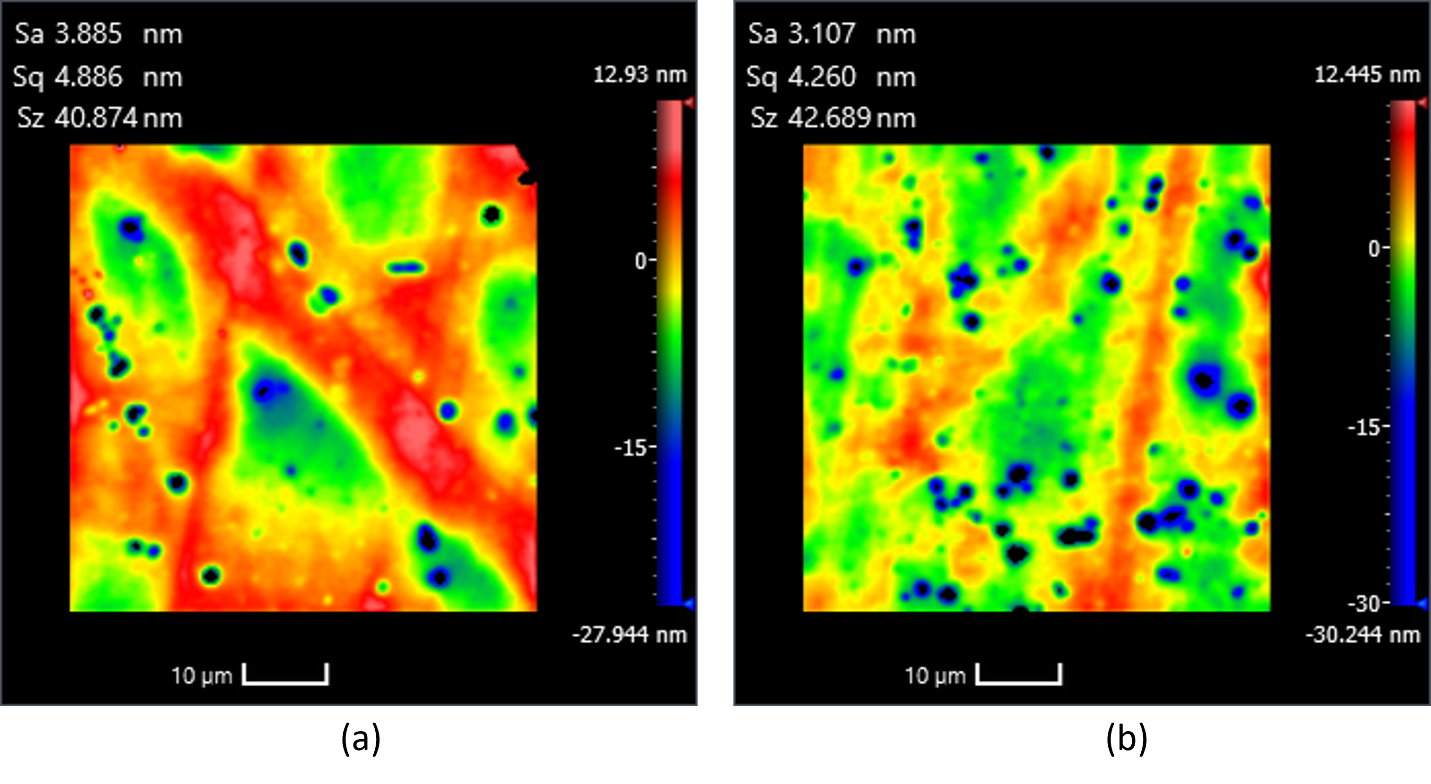


Supplementary Figure 18. The surface roughness of samples fabricated with different methods. (a) Molded lens using tetraSH-DVO2. (b) Printed lens using tetraSH-DVO2.


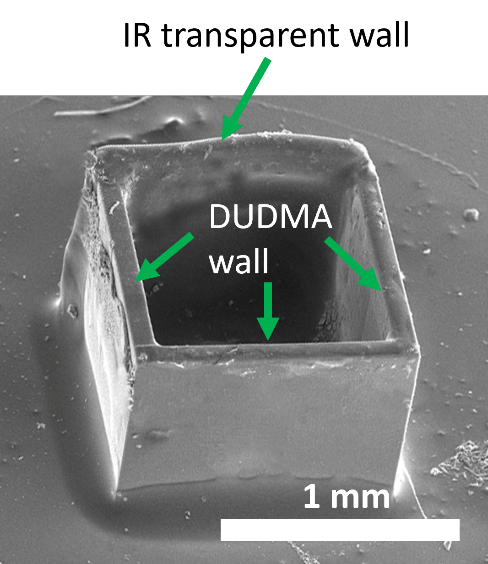


Supplementary Figure 19. 3D printed micro-reactor with 3 IR untransparent walls and 1 IR transparent wall.


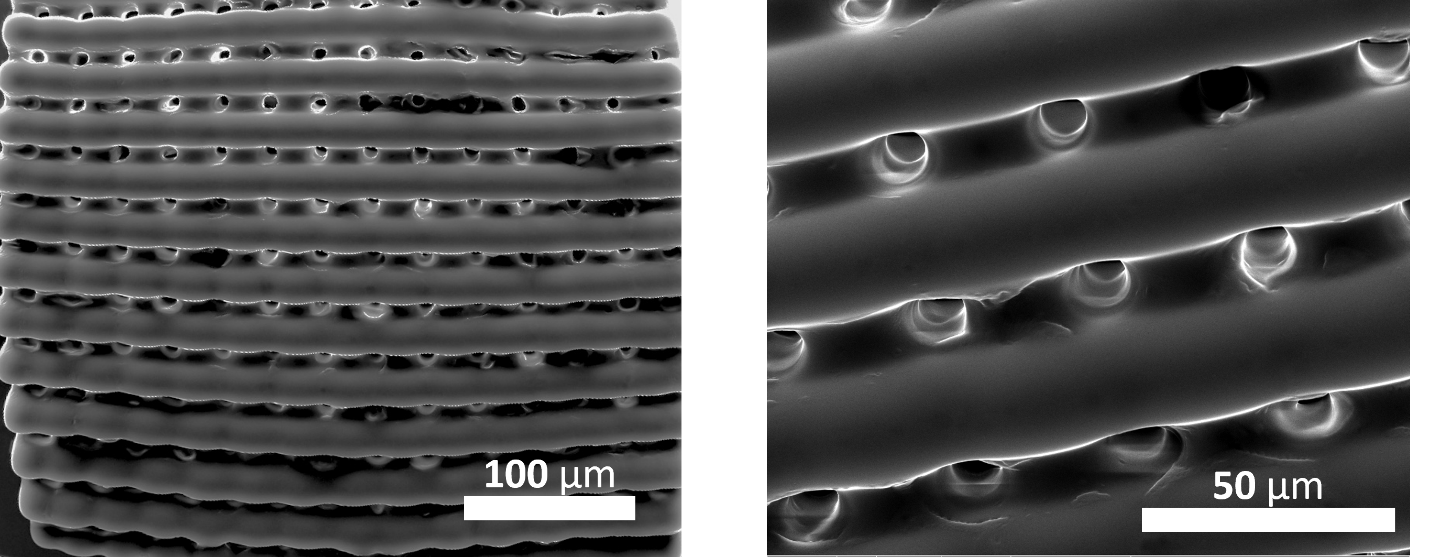


Supplementary Figure 20. 3D printed objects containing micro-channels.

**Supplementary references**

1 Geitenbeek, R. G. *et al.* Luminescence thermometry for in situ temperature measurements in microfluidic devices. *Lab on a Chip* **19**, 1236-1246 (2019). <https://doi.org:10.1039/C8LC01292J>

2 Whitesides, G. M. The origins and the future of microfluidics. *Nature* **442**, 368-373 (2006). <https://doi.org:10.1038/nature05058>

3 Chen, C. H., Chung, D. J., Bhattacharya, S., Papapolymerou, J. & Peroulis, D. in *2008 38th European Microwave Conference.* 9-12.

4 Dang, B., Bakir, M. S., Sekar, D. C., King, C. R. & Meindl, J. D. Integrated Microfluidic Cooling and Interconnects for 2D and 3D Chips. *IEEE Transactions on Advanced Packaging* **33**, 79-87 (2010). <https://doi.org:10.1109/TADVP.2009.2035999>

5 Chen, X., Mo, D. & Gong, M. 3D Printed Reconfigurable Modular Microfluidic System for Generating Gel Microspheres. *Micromachines* **11** (2020).

6 Gray, B. L. *et al.* Novel interconnection technologies for integrated microfluidic systems1Paper presented as part of the SSAW-98 Workshop.1. *Sensors and Actuators A: Physical* **77**, 57-65 (1999). <https://doi.org:https://doi.org/10.1016/S0924-4247(99)00185-5>

7 Lee, K. G. *et al.* 3D printed modules for integrated microfluidic devices. *RSC Advances* **4**, 32876-32880 (2014). <https://doi.org:10.1039/C4RA05072J>

8 Bhattacharjee, N., Urrios, A., Kang, S. & Folch, A. The upcoming 3D-printing revolution in microfluidics. *Lab on a Chip* **16**, 1720-1742 (2016). <https://doi.org:10.1039/C6LC00163G>

9 Su, R. *et al.* 3D printed self-supporting elastomeric structures for multifunctional microfluidics. *Science Advances* **6**, eabc9846 <https://doi.org:10.1126/sciadv.abc9846>

10 Lee, M. *et al.* Long-wave infrared transparent sulfur polymers enabled by symmetric thiol cross-linker. *Nature Communications* **14**, 2866 (2023). <https://doi.org:10.1038/s41467-023-38398-5>

11 Anderson, L. E. *et al.* Chalcogenide Hybrid Inorganic/Organic Polymers: Ultrahigh Refractive Index Polymers for Infrared Imaging. *ACS Macro Letters* **6**, 500-504 (2017). <https://doi.org:10.1021/acsmacrolett.7b00225>

12 Boyd, D. A. *et al.* Comonomer Isomers Result in Varied Optical Properties for Long Wavelength Infrared-Transmitting ORMOCHALC Polymers *ChemRxiv* (2023). <https://doi.org:10.26434/chemrxiv-2023-qqjkn>

13 Kleine, T. S. *et al.* Infrared Fingerprint Engineering: A Molecular-Design Approach to Long-Wave Infrared Transparency with Polymeric Materials. *Angewandte Chemie International Edition* **58**, 17656-17660 (2019). <https://doi.org:https://doi.org/10.1002/anie.201910856>

14 Boyd, D. A. *et al.* Optical Properties of a Sulfur-Rich Organically Modified Chalcogenide Polymer Synthesized via Inverse Vulcanization and Containing an Organometallic Comonomer. *ACS Macro Letters* **8**, 113-116 (2019). <https://doi.org:10.1021/acsmacrolett.8b00923>

15 Qiu, Y., Li, J., Li, T., Ma, X. & Jiang, X. Photo-Curing Vis-IR Hybrid Fresnel Lenses with High Refractive Index. *Macromolecular Chemistry and Physics* **222**, 2100311 (2021). <https://doi.org:https://doi.org/10.1002/macp.202100311>
