# Supplementary material for: UV-curable thiol-ene system for broadband infrared transparent objects
Source: Nat Commun. 2023 Dec 16;14:8385. doi: 10.1038/s41467-023-44273-0 (PMC10725491; doi:10.1038/s41467-023-44273-0)
Supplement: Supplementary file 3 — Description of Additional Supplementary Files [file 41467_2023_44273_MOESM3_ESM.docx]

**Description of Additional Supplementary Files**

Supplementary Movie 1

Description: Comparison of temperature monitoring performance between Infrared (IR) transparent thiol-ene material and conventional 3D printable resin. There are three scenes in this movie.

Scenes 1: IR transparent wall’s performance in monitoring the temperature of mixing between 5M NaOH and DI water. The thermal generated by dilution of 5M NaOH was not visible.

Scenes 2: Comparison between IR transparent wall and IR untransparent wall’s performance in monitoring the temperature of reaction between 5M NaOH and 5M HCl. It was observed that the Long-Wave Infrared (LWIR) signal caused by acid-base neutralization can pass through the IR transparent wall but cannot pass through the DUDMA wall.

Scenes 3: IR transparent wall’s performance in monitoring the temperature of reaction between NaOH particle and 5M HCl. The acid-base neutralization and the dissolving of NaOH generated strong LWIR signal that was observed.
